# Antimicrobial resistance in hospitalized surgical patients: a silently emerging public health concern in Uganda

**DOI:** 10.1186/1756-0500-6-298

**Published:** 2013-07-27

**Authors:** Jeremiah Seni, Christine F Najjuka, David P Kateete, Patson Makobore, Moses L Joloba, Henry Kajumbula, Antony Kapesa, Freddie Bwanga

**Affiliations:** 1 Department of Medical Microbiology, Makerere University College of Health Sciences, P.O. Box 7072, Kampala, Uganda; 2 Department of Surgery, Makerere University College of Health Sciences, P.O. Box 7072, Kampala, Uganda; 3 Department of Microbiology and Immunology, Catholic University of Health and Allied Sciences-Bugando, P.O. Box 1464, Mwanza, Tanzania; 4 Department of Community Medicine, Catholic University of Health and Allied Sciences-Bugando, P.O. Box 1464, Mwanza, Tanzania

**Keywords:** Antimicrobial resistance, Surgical patients, Uganda

## Abstract

**Background:**

Surgical site infections (SSIs) are difficult to treat and are associated with substantially longer hospital stay, higher treatment cost, morbidity and mortality, particularly when the etiological agent is multidrug-resistant (MDR). To address the limited data in Uganda on SSIs, we present the spectrum of bacteria isolated from hospitalized patients, the magnitude and impact of MDR bacterial isolates among patients with SSIs.

**Methods:**

A descriptive cross sectional study was conducted from September 2011 through April 2012 involving 314 patients with SSIs in the obstetrics & gynecology, general surgery and orthopedic wards at Mulago National Hospital in Kampala, Uganda. Wound swabs were taken and processed using standard microbiological methods. Clinico-demographic characteristics of patients were obtained using structured questionnaires and patients’ files.

**Results:**

Of the 314 enrolled patients with SSIs (mean age 29.7 ±13.14 years), 239 (76.1%) were female. More than half of the patients were from obstetrics and gynecology (62.1%, 195/314). Of 314 wound swabs taken, 68.8% (216/314) were culture positive aerobically, yielding 304 bacterial isolates; of which 23.7% (72/304) were *Escherichia coli* and 21.1% (64/304) were *Staphylococcus aureus*. More than three quarters of Enterobacteriaceae were found to be extended spectrum beta lactamase (ESBL) producers and 37.5% of *S. aureus* were Methicillin resistant *S. aureus* (MRSA). MDR occurred in 78.3% (238/304) of the isolates; these were more among Gram-negative bacteria (78.6%, 187/238) compared to Gram-positive bacteria (21.4%, 51/238), (p-value < 0.0001, χ2 = 49.219)*.* Amikacin and imepenem for ESBL-producing Enterobacteriacea and vancomycin for MRSA showed excellent performance except that they remain expensive drugs in Uganda.

**Conclusion:**

Most SSIs at Mulago National Hospital are due to MDR bacteria. Isolation of MRSA and ESBL-producing Enterobacteriaceae in higher proportions than previously reported calls for laboratory guided SSIs- therapy and strengthening of infection control surveillance in this setting.

## Background

Surgical site infections (SSIs) are the infections involving skin, subcutaneous tissue and organs/spaces opened or manipulated during an operation, occurring within 30 days after the procedure or within one year if orthopedic implant is in situ [1,2]. SSIs account for approximately 15.0% and rank third among all types of nosocomial infections in the USA [2,3]. Infection rates among operated patients varies with hospital settings reflecting infection control practices as well as factors related to the agent, environment and the host [4]; for example, infection rates reported were less than 3.0% in German and France, 16.9% in Rio de Janeiro, Brazil and 26.0% in Mwanza, Tanzania [5-8]. SSIs can involve any surgical procedure ranging from obstetrics & gynecology, general surgery and orthopedic [6,9,10].

The most predominant bacteria in hospital-acquired SSIs are *Staphylococcus aureus, Enterococcus spp*, *Pseudomonas aeruginosa, Escherichia coli,* and other Enterobacteriaceae; of these, single bacterial isolates are common whereas 9.0% to 27.0% of bacterial isolates from different surgical sites are attributed to polymicrobial [7-9,11]. These infections pose therapeutic challenges and are associated with substantially longer duration of hospital stay, increased hospital cost, higher morbidity and mortality [5,12], particularly when the agents are Methicillin resistant *S. aureus* (MRSA), Extended spectrum beta lactamase (ESBL) producing Enterobacteriaceae and/or other agents collectively referred to as multidrug-resistant (MDR) [11,13,14]. Studies from developing countries have shown high level of resistance (ranging from 50 to 100%) to the commonly used antibiotics like ampicillin, trimethoprim – sulphamethoxazole, gentamicin, chloramphenicol and third generation cephalosporins among *S. aureus, E. coli*, and *P. aeruginosa*[8,15] as opposed to low rates of resistance ranging from 0-50% in developed countries [16]. In both settings however, substantial rates of resistance to oxacillin, erythromycin and clindamycin reported for *S. aureus,* ranged from 10-60% [8,9,15,16] whereas vancomycin (for *S. aureus* and other Gram-positive bacteria*),* amikacin, piperacillin-tazobactam and imepenem (for *E. coli*, *P. aeruginosa* and other Gram-negative bacteria) showed resistant rates of less than 25% [17,18].

It is well known that specific therapeutic options to patients with SSIs are largely dependent on data from antimicrobial sensitivity tests generated by clinical laboratories or sound epidemiological data from ongoing nosocomial infection surveillance [6,11,19].

In Uganda, about 10% of the surgical procedures become septic accounting for an increasing morbidity and mortality, with the commonest organism isolated being *S. aureus*[20-22]; however data on the spectrum of bacteria isolated from hospitalized patients and their antimicrobial susceptibility patterns to guide SSI-therapy in Mulago National Hospital remains scanty. Furthermore, the magnitude and impact of MDR bacteria from SSIs are unknown. Thus, this study aimed at addressing these areas. Data herein will be crucial in guiding SSIs-therapy and will form a baseline for nosocomial SSIs surveillance.

## Methods

### Study design and sampling process

This descriptive cross-sectional study was conducted at Mulago National Hospital in Kampala, Uganda. The hospital is located on Mulago Hill in the northern part of the city of Kampala and is the largest hospital in Uganda with an estimated 1,500 beds.

The study was conducted for a period of 8 months from September 2011 to April 2012 and involved 314 patients with clinical SSIs who consented to participate. The patients were from obstetrics & gynecology, general surgery and orthopedic wards. All patients with SSIs occurring within 30 days after the operative procedure or within one year if orthopedic implant was in situ were included, whereas surgical patients with community-acquired pyogenic infections such as abscess, furuncle and carbuncles; patients with infection of an episiotomy; and patients with open fractures were excluded from the study.

### Study clearance and ethical considerations

The study got ethical clearance from the Institutional Review Board (IRB) of Makerere University College of Health Sciences (# REC REF 2011–183), Mulago Hospital Research Committee (MREC #125) and the Uganda National Council for Science and Technology (UNCST) (REF # HS 1080). A written informed consent from each patient/caretaker and assent for minors (11 to 17years) were obtained whereas for each minor (<11 years), consent was obtained from his/her parent or caretaker. All patient information was kept confidential and anonymous using codes.

### Data collection and laboratory procedures

Demographic and clinical characteristics from patients were collected using structured questionnaire and from patients’ files (see Additional file 1). The infected site was cleaned using normal saline and sterile gauze then, from each patient, two wound swabs were collected using sterile cotton swabs in Amies transport media (Biolab, HUNGARY®).

#### Isolate identification

Wound swabs were processed in the bacteriology laboratory of the Department of Medical Microbiology, Makerere University College of Health Sciences, within 2 hours of collection. The first wound swab was used to make Gram stain smears while the second one was inoculated into blood agar, MacConkey agar, and mannitol-salt agar and incubated at 35-37°C for 24–48 hours. Identification of bacteria was based on conventional physiological and biochemical methods such as Gram stain, catalase reaction, coagulase test, DNase test, hemolytic activity on sheep blood agar plates, bacitracin, optochin and trimethoprim-sulphamethoxazole (SXT) antimicrobial identification disks and bile esculin test for Gram-positive bacteria. Gram-negative bacteria were identified based on colony morphology on blood agar and MacConkey agar, followed by biochemical reactions namely oxidase, triple sugar iron (TSI), sulphur indole and motility (SIM), citrate, and urease tests [23].

#### Drug susceptibility tests

Following identification of the bacterial isolates, a standard disc diffusion technique for drug susceptibility test (DST) was performed as recommended by Clinical and Laboratory Standard Institute (CLSI) [24]. For Gram-positive bacteria, discs (Biolab®, HUNGARY) tested were ampicillin (10 μg), oxacillin (1 μg), trimethoprim-sulphamethoxazole (1.25/23.75 μg), tetracycline (30 μg), ciprofloxacin (5 μg), chloramphenicol (30 μg), gentamicin (10 μg) [high level gentamicin (120 μg) for *Enterococcus spp*], erythromycin (15 μg), clindamycin (2 μg), and vancomycin (30 μg). For Gram-negative bacteria discs (Biolab®, HUNGARY) tested included ampicillin (10 μg), piperacillin(100 μg), piperacillin-tazobactam (100/10 μg), amoxicillin-clavulanic acid (20/10 μg), trimethoprim-sulphamethoxazole (1.25/23.75 μg), tetracycline (30 μg), ciprofloxacin (5 μg), chloramphenicol (30 μg), gentamicin (10 μg), amikacin (30 μg), ceftriaxone (30 μg), ceftazidime (30 μg), cefepime (30 μg), and imipenem (10 μg). These were incubated at 35-37°C for 24 hours.

Isolates which were not identifiable by the standard conventional methods, colistin DST for all *Acinetobacter spp* and *P. aeruginosa* as well as vancomycin DST for all *S. aureus* were confirmed using the Phoenix Automated instrument® (Becton-Dickson, Sparks Maryland) as per manufacturer’s instruction.

For determining inducible clindamycin resistance, clindamycin disk (2 μg) and erythromycin disk (15 μg) were placed side by side approximately 15-26 mm apart. Flattening of the zone of inhibition adjacent to the erythromycin disk was regarded as a positive D-test. As recommended by CLSI, isolates were screened for ESBL production using the double disc method and MRSA was identified by the use of cefoxitin disc (30 μg) [24,25]. MDR was defined as an isolate with resistance to three or more antimicrobial classes [26].

Results on isolate identity and antimicrobial susceptibility patterns were promptly reported to the attending doctor for patient care.

### Quality control

Reference strains *S. aureus* ATCC 25923 and *Staphylococcus epidermidis* ATCC 12228 for Gram-positive bacteria and *E. coli* ATCC 25922 and *P. aeruginosa* ATCC 27853 for Gram-negative bacteria were used to quality-control microbiological procedures such as Gram staining , growth of bacteria on respective media, microscopy, biochemical identification tests and drug susceptibility testing.

### Data analysis

Variables from the clinical and demographic data in the questionnaire and laboratory data were entered into Excel®, cleaned and exported to STATA software version 11 (College Station, Texas, USA) for analysis according to the objectives of the study. Continuous variables were described as mean (± standard deviation). Categorical variables were described as proportion and were analyzed to compare the significance of difference in distribution by using Chi square test or Fischer’s exact test where appropriate. To determine factors associated with bacteria isolation from SSI, we used univariate followed by multivariate logistic regression analysis. At univariate level all factors which had a *p-*value of less than 0.05 were subjected to multivariate analysis. The strength of association between factors and outcome was measured using odds ratio with respective 95% confidence interval. Factors with *p-*value of less than 0.05 on multivariate logistic regression analysis were considered as independent association of bacteria isolation from SSI.

## Results

This study enrolled 314 patients with clinical SSIs. Among these, 239 (76.1%) were female. The overall mean age was 29.7 ± 13.14 years (minimum 12 and maximum 83 years). More than half of the patients were from obstetrics and gynecology wards, 62.1% (195/314), whereas 33.1% (104/314) and 4.8% (15/314) were from general surgery and orthopedic wards respectively. The most common surgical procedures were caesarean section 46.2% (145/314) and laparotomy 42.7% (134/314); open reduction and internal fixation (ORIF) accounted for 3.5% (11/314) while other surgical procedures contributed 7.6% (24/314).

**Figure 1:**
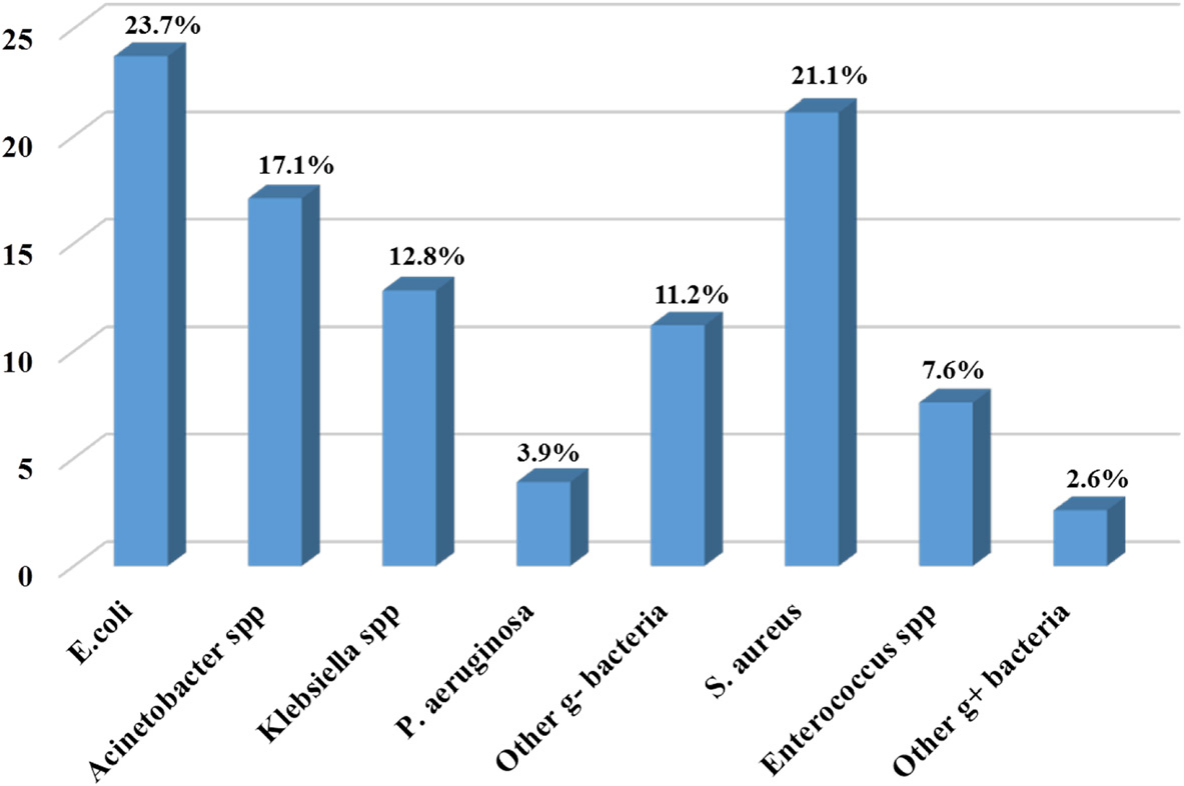
**Proportion of bacterial isolates from patients with surgical site infections (N = 304).** Other g- bacteria: *Enterobacter cloacae* (10)*, Proteus mirabilis* (7)*, Morganella morganii* (6)*, Providencia spp* (5), *Citrobacter freundii* (4), *Serratia marcescens* (1) and *Leclercia adecarboxylata* (1); Other g + bacteria: *Streptococcus pyogenes* (3), *Streptococcus agalactiae* (1), and *Streptococcus spp* (4).*Acinetobacter spp*: *Acinetobacter baumanii* (48) and *Acinetobacter baumanii-calcoaceticus complex* (4); *Klebsiella spp*: *Klebsiella pneumonia* (35) and *Klebsiella oxytoca* (4); *Enterococcus spp*: *Enterococcus faecalis* (21) and *Enterococcus faecium* (2).

Of the 314 non-repeat wound swabs collected, 216 (68.8%) were culture positive aerobically. The most predominant bacterial isolates were *E. coli,* 23.7% (72/304) and *S. aureus,* 21.1% (64/304) (see Figure 1). Single bacterial isolates were recovered from 137 (63.4%) patients whereas 79 (36.6%) had polymicrobial infections.

All *P. aeruginosa* isolates were sensitive to colistin, whereas 3.9% (2/52) of *Acinetobacter spp* were resistant to colistin. All Gram-negative bacteria were susceptible to imipemen, except 3.9% (2/52) of *Acinetobacter spp*. All *S. aureus* isolates were sensitive to vancomycin, whereas one *Enterococcus spp* [4.4% (1/23)] was resistant to vancomycin i.e. VRE (see Table 1).

**Table 1:** Antimicrobial resistance pattern among isolates from surgical site infections

**Antibiotics**	**Bacteria isolates (N = 304)**							
	***E.coli *(n = 72)**	***Klebsiella spp *(n = 39)**	***Acinetobacter spp *(n = 52)**	***P.aeruginosa *(n = 12)**	**Other g- (n = 34)**	***S. aureus *(n = 64)**	***Enterococcus spp *(n = 23)**	**Other g + (n = 08)**
**Ampicillin**	100.0%	100.0%	NA	NA	97.1%	100.0%	30.0%	50.0%
**Piperacillin**	NA	NA	100.0%	66.7%	NA	NA	NA	NA
TZP^*^	30.6%	38.5%	53.8%	16.7%	32.4%	NA	NA	NA
**AMC**^**+**^	90.3%	100.0%	NA	NA	94.1%	NA	NA	NA
**TMP-SMX **^**×**^	86.1%	92.3%	98.0%	100.0%	94.1%	89.1%	NA	80.0%
**Tetracycline**	72.2%	76.9%	65.4%	100.0%	82.4%	42.2%	74.0%	62.5%
**Ciprofloxacin**	72.2%	66.6%	77.0%	16.7%	47.1%	29.7%	60.9%	50.0%
**Chloramphenicol**	41.7%	71.8%	NA	NA	70.6%	15.6%	30.4%	37.5%
**Gentamicin**	54.2%	76.9%	88.5%	16.7%	76.5%	18.8%	21.7%^**^	50.0%
**Amikacin**	4.2%	2.6%	32.7%	8.3%	0.0%	NA	NA	NA
**Ceftriaxone**	77.8%	92.3%	NA	NA	67.6%	NA	NA	NA
**Ceftazidime**	77.8%	87.2%	90.4%	25.0%	61.8%	NA	NA	NA
**Cefepime**	69.4%	87.2%	61.5%	16.7%	38.2%	NA	NA	NA
**Imipenem**	0.0%	0.0%	3.9%	0.0%	0.0%	NA	NA	NA
**Erythromycin**	NA	NA	NA	NA	NA	46.9 %	65.2%	50.0%
**Clindamycin**	NA	NA	NA	NA	NA	40.6%	NA	62.5%
**Vancomycin**	NA	NA	NA	NA	NA	0.0%	4.4%	0.0%

^*^Piperacillin-tazobactam, ^**+**^Amoxicillin-clavulanate, ^×^Trimethoprim-sulphamethoxazole, ^**^ High level resistance screening, *NA*: Not applicable, *spp*: species, *g+*: Gram-positive bacteria [*Streptococcus pyogenes* (3), *Streptococcus agalactiae* (1), and *Streptococcus spp* (4)] and *g-*: Gram-negative bacteria [*Enterobacter cloacae* (10)*, Proteus mirabilis* (7)*, Morganella morganii* (6)*, Providencia spp* (5), *Citrobacter freundii* (4), *Serratia marcescens* (1) and *Leclercia adecarboxylata* (1)].

More than three quarters of Enterobacteriaceae were phenotypically found to be ESBL producers [*E. coli*, 79.2% (57/72); *Klebsiella spp,* 92.3% (36/39) and other Enterobacteriaceae, 73.5% (25/34)]. The proportion of MRSA among *S. aureus* isolates was 37.5% (24/64).

Of the 304 isolates recovered from SSIs, 78.3% (238/304) were MDR, these were found significantly more among Gram-negative bacteria (78.6%, 187/238) compared to Gram-positive bacteria (21.4%, 51/238) (p-value < 0.0001, χ2 = 49.219).

In the univariate analysis, bacterial isolation from SSIs among patients was associated with age greater than 40 years, sex, ward type, operation (whether elective or emergency), duration from admission to operation, duration from admission to discharge, type of antibiotic chemoprophylaxis given, duration of postoperative antibiotics and outcome (see Table 2).

**Table 2:** Association of clinico-demographic characteristics of patients with and without bacterial isolates from surgical site infections

**Variable**		**Bacteria isolated from SSI (n = 216)**	**No bacteria isolated from SSI (n = 98)**	**OR [95% CI]**	**P-value**
**Age (years)**	**≤ 19**	38 (67.9%)	18 (32.1%)	1	
	**20 – 39**	133 (63.6%)	76 (36.4%)	0.83(0 .42- 1.61)	0.5577
	**40 – 59**	31 (91.2%)	3 (8.8%)	4.89( 1.24- 27.87)	0.0112
	**≥ 60**	14 (93.3%)	1 (6.7%)	6.63( 0.86- 295.67)	0.0478
**Sex**	**Male**	61 (81.3%)	14 (18.7%)	1	
	**Female**	155 (64.9%)	84 (35.1%)	0.42(0 .21- 0.82)	0.0072
**Ward type**	**Obst & Gyn**	115 (59.0%)	80 (41%)	1	
	**General Surgery**	89 (85.6%)	15 (14.4%)	4.13( 2.17- 8.22)	0.0000
	**Orthopedic**	12 (80.0%)	3 (20.0%)	2.78(0.72- 15.79)	0.1085
**Operation**	**Elective**	57 (83.8%)	11 (16.2%)	1	
	**Emergency**	159 (64.6%)	87 (35.4%)	0.35( 0.16- 0.73)	0.0025
**Duration from admission to operation**	**≤ 1 day**	147 (65.3%)	78 (34.7%)	1	
	**> 1 days**	69 (77.5%)	20 (22.5%)	1.83( 1.01- 3.42)	0.0356
**Duration from admission to discharge**	**≤ 14days**	48 (58.5%)	34 (41.5%)	1	
	**> 14days**	110 (82.7%)	23 (17.3%)	3.39( 1.73- 6.68)	0.0001
**Antibiotic chemoprophylaxis**	CRO^*^	124 (62.9%)	73 (37.1%)	1	
	CRO^*^ + MZ^**^	70 (76.9%)	21 (23.1%)	1.96( 1.08- 3.65)	0.0187
	**Others**^**×**^	22 (84.6%)	4 (15.4%)	3.24( 1.04- 13.37)	0.0289
**Duration of Postoperative antibiotics**	**≤ 3days**	48 (50.5%)	47 (49.5%)	1	
	**> 3days**	168 (76.7%)	51 (23.3%)	3.23( 1.87- 5.54)	0.0001
**Outcome**	**Improved**	147 (72.1%)	57 (27.9%)	1	
	**Died**	10 (100.0%)	0 (0.0%)	-	0.042

^*^Ceftriaxone, ^**^Metronidazole, ^**×**^Ampicloxacillin, Ciprofloxacin & Gentamicin.

On multivariate logistic regression analysis, longer duration from admission to discharge, longer duration of postoperative antibiotics and outcome were statistically associated with bacteria isolation from SSIs (see Table 3). Of ten patients who died, 70% (7/10) had ESBL producing Enterobacteriaceae, four of which were *E. coli.*

**Table 3:** **Multivariate logistic regression analysis for factors associated with bacterial isolation from SSI**s

**Variable**	**OR**	**95% CI**	**p-value**
Age (years)	1.16	0.74 - 1.80	0.524
Sex	1.41	0.49 - 4.02	0.522
Wards	0.44	0.18 - 1.10	0.078
Operation	0.62	0.24 - 1.65	0.343
Duration from admission to operation	0.89	0.40 - 1.97	0.773
Duration from admission to discharge	1.93	1.01 - 3.72	0.047
Antibiotic chemoprophylaxis	1.27	0.77 - 2.12	0.351
Duration of postoperative antibiotics	2.52	1.45 - 4.36	0.001
Outcome	0.52	0.30 - 0.91	0.021

## Discussion

Of the 314 patients with clinical SSIs enrolled in this study; the mean age (29.7 ± 13.14 years), higher female proportion (76.1%) and preponderance of admission in obstetrics and gynecology wards (62.1%) was similar to another study [11], but the proportion of female in other studies were between 30% to 60% and wards of admission varied reflecting the underlying surgical procedures [8,9,13]. Caesarean section and laparotomy accounted for more than three quarters of all surgical procedures in this study whereas other similar studies have also found these procedures to be quite common [8,11,27]. The predominance of SSIs cases in obstetrics and gynecology wards is quite alarming and thus, a need to institute stringent infection prevention and control measures in this setting, more especially in emergency surgeries which accounted for more SSIs cases as opposed to elective surgeries.

As noted from other studies [8,9,11,28], *S. aureus* and *E. coli* were the most common bacterial isolates from SSIs. However their sources remain unknown from the present study though other studies have documented both endogenous and exogenous sources from hospital environment could be potential niches [29,30]. Similar to other related studies [9,18,31], high level of resistance was found among commonly used antibiotics like ampicillin, trimethoprim-sulphamethoxazole, and tetracycline in both Gram-positive and Gram-negative bacteria. Gram-negative bacteria showed more resistance to gentamicin, ciprofloxacin and chloramphenicol as compared to Gram-positive bacteria. With exception of *Acinetobacter spp*, all Gram-negative bacteria displayed low resistance rates to piperacillin-tazobactam, amikacin and imipenem. There were also low resistance rates of *Acinetobacter spp* and *P. aeruginosa* to colistin. These findings are similar to another study [17]. The present study has shown that the rates of resistance to erythromycin (46.9%) among *S. aureus* was relatively low compared to that of *Enterococcus spp* (65.2%), with excellent performance of vancomycin on both *S. aureus* and *Enterococcus spp*. These findings are in agreement to another study [18]. This study found more MDR among Gram-negative bacteria than Gram-positive bacteria; of these the proportion of MRSA (37.5%) among *S. aureus* isolates was more than previously reported (25.0% and 31.5%) from Uganda [9,21] and other countries [8,16,18]. Thus, while β-lactamase-resistant antibiotics such as cloxacillin could still be effective in this setting, they are likely to be ineffective against the 38% of isolates that were confirmed as MRSA. The fact that we found no vancomycin resistance among *S. aureus* isolates shows that this drug remains the last resort in systemic infections caused by MRSA in this setting. Previous studies [11,17] have shown low rates (14% to 22%) of ESBL-producers among Enterobacteriaceae isolates but the present study and another similar study in the same region [8] have shown ESBL-producers to account more than three quarter of Enterobacteriaceae. This can be attributed to the empirical use of third generation cephalosporins (usually in combination with another drug such as gentamicin) in almost all hospitalized patients and lack of antimicrobial resistance surveillance in surgical wards at Mulago National Hospital. This is a major threat to patient care as ESBL production renders use of these ceftriaxone or ceftazidime useless. Absence of resistance to imepenem among these isolates is however a good finding, except that imepenem remains an expensive drug.

Multivariate logistic regression analysis of clinical and demographic characteristics of patients with SSIs in this study showed that longer duration from admission to discharge, longer duration of postoperative antibiotics and outcome (death) were associated with bacteria isolation from SSIs. These findings have also been shown in other similar studies [5,11,27].

### Limitation

The study did not isolate strict anaerobes, which could have increased the number of bacterial isolates currently reported as negative cultures. This was because of lack of standardized in-house detection methods and lack of anaerobic detection panels in the Phoenix Automated instrument (Becton-Dickson, Sparks Maryland) that we used.

## Conclusion

Most SSIs at Mulago National Hospital are due to MDR bacteria, these are significantly more among Gram-negative than Gram-positive bacteria. Isolation of MRSA and ESBL-producing Enterobacteriaceae in higher proportions than previously reported calls for enhanced antibiotic stewardship including laboratory guided SSIs-therapy and strengthening of infection control surveillance by identifying sources of these MDR isolates. In the light of these findings, there is a need to investigate whether there is clonal spread of the predominant bacteria within/or among surgical wards at Mulago National Hospital.

## Competing interests

The authors declare that they have no competing interests.

## Authors’ contributions

Conceived and designed the experiments: JS, DPK, and FB. Specimen collection: JS. Supervised the clinical component of research: PM. Performed the experiments: JS. Supervised the laboratory component of research: CFN, DPK, and FB. Analyzed the data: JS, DPK, AK, and FB. Contributed reagents, materials and analysis tools: JS, CFN, HK, and MLJ. Wrote the manuscript: JS, CFN, DPK, PM, MLJ, HK, AK, and FB. All authors have read and approved the final manuscript.

## Supplementary Material

Additional file 1 **Appendix i.** Questionnaire. Click here for file
