# Supplementary material for: Antimicrobial resistance in hospitalized surgical patients: a silently emerging public health concern in Uganda
Source: BMC Res Notes. 2013 Jul 27;6:298. doi: 10.1186/1756-0500-6-298 (PMC3729663; doi:10.1186/1756-0500-6-298)
Supplement: Additional file 1 — Appendix i. Questionnaire. [file 1756-0500-6-298-S1.doc]

**Appendix i: Questionnaire**

1. Patient Code number ………………………….
2. Sex ………………….M/F
3. Age …………………years
4. Ward………………...........
5. Date of admission…………..
6. Duration of onset in the hospital…………days
7. Surgical site infection:
8. Preoperative diagnosis……………………
9. Type of operation………………………...
10. Elective / Emergency…………………….
11. Antibiotic chemoprophylaxis given preoperative …………..........
12. Duration from admission to operation……(hours/days)
13. SSI detected on how many days postoperative ………………….
14. SSI classification (a) Superficial SSI (b) Deep SSI (c) Organ SSI
15. Length of hospital stay before index culture…………………………
16. Antibiotics used post-operatively before index culture taken
17. Type………………………………..Duration……...days
18. Type………………………………..Duration………days
19. Type………………………………..Duration……….days
20. Outcome:
21. Improved and discharged………… days in hospital stay.
22. Died………………………………...days to death.
